# Predicting Drug Release Rate of Implantable Matrices and Better Understanding of the Underlying Mechanisms through Experimental Design and Artificial Neural Network-Based Modelling

**DOI:** 10.3390/pharmaceutics14020228

**Published:** 2022-01-19

**Authors:** Ernő Benkő, Ilija German Ilič, Katalin Kristó, Géza Regdon, Ildikó Csóka, Klára Pintye-Hódi, Stane Srčič, Tamás Sovány

**Affiliations:** 1 Institute of Pharmaceutical Technology and Regulatory Affairs, University of Szeged, Eötvös u. 6, H-6720 Szeged, Hungary; benko.erno.mate@egis.hu (E.B.); kristo.katalin@szte.hu (K.K.); geza.regdon@pharm.u-szeged.hu (G.R.J.); csoka.ildiko@szte.hu (I.C.); klara.hodi@pharm.u-szeged.hu (K.P.-H.); 2 Department of Pharmaceutical Technology, University of Ljubljana, Aškerčeva cesta 7, SI-1000 Ljubljana, Slovenia; ilija.german.ilic@ffa.uni-lj.si (I.G.I.); stanko.srcic@ffa.uni-lj.si (S.S.)

**Keywords:** drug–excipient interaction, polymers, nondegradable, matrix tablet, controlled release, design of experiments, artificial neural networks

## Abstract

There is a growing interest in implantable drug delivery systems (DDS) in pharmaceutical science. The aim of the present study is to investigate whether it is possible to customize drug release from implantable DDSs through drug–carrier interactions. Therefore, a series of chemically similar active ingredients (APIs) was mixed with different matrix-forming materials and was then compressed directly. Compression and dissolution interactions were examined by FT-IR spectroscopy. Regarding the effect of the interactions on drug release kinetics, a custom-made dissolution device designed for implantable systems was used. The data obtained were used to construct models based on artificial neural networks (ANNs) to predict drug dissolution. FT-IR studies confirmed the presence of H-bond-based solid-state interactions that intensified during dissolution. These results confirmed our hypothesis that interactions could significantly affect both the release rate and the amount of the released drug. The efficiencies of the kinetic parameter-based and point-to-point ANN models were also compared, where the results showed that the point-to-point models better handled predictive inaccuracies and provided better overall predictive efficiency.

## 1. Introduction

Matrix tablets belong to the most popular systems for controlled delivery of drugs. Drug liberation from porous matrices is a complex process, influenced by numerous parameters such as solubility, matrix porosity [1], swelling [2] and gel formation [3] of polymers. The gel properties are influenced by particle size [1,4] or by the molecular weight of polymers [5] and they generally determine the drug transport within the matrix, which may be diffusion-driven [2], relaxation-driven [6], external mass-transport-driven or anomalous [7]. Especially, the anomalous transport mechanism presupposes the presence of interparticle interactions within the system [5,7]. Novel achievements require the reconsideration of traditional thinking about inert excipients, and they turn the investigation and utilization of drug–excipient interactions into a focus of the development of tailored drug delivery systems.

Most of the polymers used as pharmaceutical excipients can form supramolecular conjugates [8], and the resulting H-bond-based, ionic drug excipient [9], or excipient–excipient [10] interactions may provide an ideal way to customize drug release. The formation of supramolecular interactions is most likely in dissolution or melt-based production processes, but de Robertis et al. described the occurrence of similar interactions in directly compressed systems [11]. In our previous study, we examined the release of risedronate sodium from biodegradable and non-degradable implants. Although the slowest release rate has been shown in low porosity non-degradable systems, unexpectedly prolonged, sustained release was observed in the degradable systems even after complete matrix disintegration. This was due to a strong H-bond-based interaction between risedronate sodium and chitosan, suggesting that the role and presence of intermolecular interactions in the drug release rate of directly compressed matrices could be significantly underestimated [12].

Therefore, the primary goal of the present study is to confirm the hypothesis that drug–excipient interactions can be used to further prolong drug release from non-degradable matrices, while the strength of the interactions can be predicted based on the acid strength of the drugs.

To confirm the primary hypothesis, the original PVC matrix was combined with Eudragit of different grades as a model excipient, which allowed for the development of drug-binder interactions, which are mentioned as a favourable choice to produce controlled-release DDSs. Weak base Eudragit E100 and acidic Eudragit L100-55 are commonly used for oral administration, but recent reports [13,14,15,16,17,18,19] have investigated their applicability in parenteral drug administration. Following intramuscular administration of Eudragit-based nanoparticles, safety tests were performed [18] to examine liver and skeletal muscle damage during the 6-week experiment. Tissue samples from the liver were similar to the control group; skeletal muscle showed inflammation in the first week, which decreased by the second week; at the end of the experiment, it became similar to the control group as well. In the case of intravenous microparticulate administration [13], only local inflammation was observed. Overall, the use of these materials as implantable excipients is considerable, especially because quaternerised Eudragit can facilitate paracellular API permeation at pH 7.4, which highlights the benefits of Eudragit copolymers.

A quality by design based approach was used to investigate the effect of critical material attributes on the mechanical properties of the matrices, the strength of the interaction, and the drug release rate. Design of experiments (DoE) was used to reveal basic relationships, while artificial neural networks (ANN) were used for the prediction of drug dissolution of the designed matrices. ANNs are commonly used types of machine learning/deep learning methods which mimic the signal transduction and learning mechanism of the human brain. ANNs can self-accommodate to the learning environment and are therefore widely applied for modelling of difficult nonlinear problems in a wide range of applications, including pharmaceutics. Previous studies [20,21] have demonstrated that ANNs are excellent tools for modelling the mechanical properties of tablets, whereas Galata et al. had successfully used ANNs to model drug release from hydrophilic, sustained-release matrix systems [22] by point-to-point modelling of drug release. Nevertheless, the question of how the developed network may be used for the modelling of general problems remained open. A secondary objective of the present study was to reveal if the tested material attributes will enable a generalized prediction of drug dissolution, and furthermore, another objective was to compare the efficiency of point-to-point modelling with another method where kinetic parameters (shape parameters of the dissolution curve) served as output variables.

## 2. Materials and Methods

### 2.1. Materials

Aceclofenac (ACE) was a kind gift from ExtractumPharma Ltd. (Budapest, Hungary), while diclofenac sodium (DIS), paracetamol (PAR) and polyvinyl chloride (polymerization degree: 60–150) (PVC) were kindly gifted by Gedeon Richter Plc. (Budapest, Hungary). A powder form of Eudragit E100, Eudragit EPO (EE) and Eudragit L100-55 (EL) was supplied by Evonik Industries AG (Essen, Germany). The main physicochemical properties of raw materials are summarized in Table 1.

### 2.2. Methods

#### 2.2.1. Tablet Preparation

Tablets were prepared according to a mixed 2- and 3-level factorial design, where the acidic or basic nature of excipients (e.g., the use of Eudragit E or L) was studied as 2-level, while the acidic strength of API, the weight ratio of excipients and the applied compression force were studied as 3-level factors. The detailed experimental plan is shown in Table 2.

Powder mixing was made with a Paul Schatz principle based 1.5 L mixer (Inversina, BioEngineering, Wald, Switzerland) at 45 rpm for 8 min and then for another 2 min with the addition of 1% magnesium stearate as lubricant. The mixtures were compressed with an instrumented Kilian SP 300 hydraulic press (IMA Kilian GmbH & Co., KG, Cologne, Germany) using 7 mm-diameter punches with a bevelled edge. The mixtures were loaded into the die manually then compressed in a semi-automatic ’jogging’ mode with the application of 2.9, 8.7, or 14.4 kN compression force (75, 225 and 375 MPa compression pressure, respectively).

#### 2.2.2. Physical Properties

The physical characterization of tablets was made with a Kraemer UTS tablet tester (Kraemer Elektronik GmbH, Germany). Mass, hardness, thickness and diameter were measured. The true density of matrices (*ρ_true_*) was determined with a helium gas pycnometer (AccuPyc 1330, Micromeritics, Norcross, GA, USA), while apparent density (*ρ_app_*) was calculated from their mass and physical dimensions. Then, porosity (ε) was obtained according to the following equation (Equation (1)):

*ε* = 1− (*ρ_app_*/*ρ_true_*) (1)

#### 2.2.3. Physicochemical Characterization

For the specific identification and characterization of interactions, FT-IR spectra were acquired. ZeSe HATR accessory was used, and measurements were taken with a resolution of 4 cm^−1^, a scan number of 128, with CO_2_ and H_2_O correction. Spectral data were evaluated with SpectraGryph software v1.2.10 (Dr. F. Menges, Berchtesgaden, Germany).

#### 2.2.4. Dissolution Tests

According to the requirements of implantable matrix systems, a custom-made dissolution tester was applied, imitating the dissolution environment of the tissue matrix and the shearing properties of other flow-through equipment [13]. Tablets were placed into Erlenmeyer flasks containing 50 mL of pH 7.4 phosphate buffered saline solution. The dissolution medium was circulated with an Alitea-XV (Alitea, Sweden) peristaltic pump using a flow rate of 2 mL/min. Due to the vast number of samples to be studied, 24 h dissolution tests were conducted for all compositions to obtain the most important kinetic parameters, and only those samples were selected afterwards for a one-week study in which the lowest release rates were observed. The concentration gradient was continuously maintained by mimicking the time related systemic renewing of the body fluids. Therefore, in the 24 h long experiments 2.5, 5, 5, 5, 10, and 20 mL samples were taken and replaced with fresh medium after 15 and 30 min and 1, 2, 4, and 8 h, respectively, while the last samples were taken after 24 h. In the case of the one-week study, 5, 5, 10, 20, 35, 35, and 35 mL volumes were sampled and refreshed as above after 1, 2, 4, 8, 24, 48, and 72 h, respectively, while the last samples were taken after 168 h. From each composition, three parallel measurements were taken. Quantitative analysis was made with a ThermoGenesys UV-spectrometer at a wavelength of 274 nm for aceclofenac, 276 nm for diclofenac sodium and 244 nm in the case of paracetamol. The dissolution kinetics was characterized according to the Korsmeyer–Peppas equation (Equation (2)).

*M_t_*/*M*_0_ = *kt^n^* (2)

where *M_0_* is the initial drug amount in the matrix, *M_t_* is the drug amount at the given time (*t*), *k* is the dissolution rate constant and *n* is the release exponent regarding the diffusion mechanism.

#### 2.2.5. Design of Experiments and Artificial Neural Networks

DoE analysis and ANN modelling were performed with Statistica v.13.5.0.17 (Tibco Software Inc., Palo Alto, CA, USA). Compression pressure (x_1_), amount of excipient (x_2_), API (x_3_) and excipient used (x_4_) were examined as independent factors, while hardness (y_1_), porosity (y_2_) and release rate (y_3_) were used as optimization parameters in the DoE analysis.

The secondary objective of the present study was to compare the effectiveness of various modelling approaches to enable generalized prediction of drug dissolution rates from different matrices. In kinetic-based modelling, the release rate and release exponent were used as the output of the ANN model, while in point-to-point modelling, the dissolved amount of drug at the various sampling times was applied according to Galata et al. [22], which included 7 data points. Our hypothesis is that kinetic-based modelling allows for a simplified network structure and faster generalization. To clarify the importance of different input parameters for predictive accuracy, 3 different parameter combinations were used to train the ANNs for both kinetic based and point-to-point modelling. A list of inputs used in the different training approaches is provided in Table 3, while a detailed data set for training, testing, and validating ANNs is provided in Table S1.

Feed-forward, back-propagation multilayer perceptron networks were developed in all cases. The networks were trained with BFGS algorithm. The number of hidden neurons was gradually increased in a dynamic system depending on the number of input and output neurons: *I + O − 3 ≤ n ≤ I + O + 1*, where *I* is the number of inputs *O* is the number outputs and *n* is number of hidden neurons; thus, the hidden neuron number varied from 4–8, 6–10, 8–12, 9–13, 11–15, 13–17 in cases of approach 1–6, respectively.

A multistart method including 10,000 networks was applied using the automated neural networks module of Statistica with each hidden neuron number, including a training approach to screen the best performing network with different initialization patterns and activation functions for hidden and output neurons. The training was stopped when the root mean square error (RMSE) of test the dataset reached its minimum. The 5 best performing networks from each multistart run were retained for further analysis.

The prediction performance of the networks was evaluated based on network perfection, which is the mean R^2^ of the observed vs. predicted data of each output neuron, and on the RMSE of predictions on the validation subset.

## 3. Results and Discussion

### 3.1. Physical Parameters

The physical parameters of the tablets are shown in Table 4, while the statistical evaluation of the effect of compression pressure (x_1_), amount of excipient (x_2_), API (x_3_) and excipient used (x_4_) on hardness (y_1_) and porosity (y_2_) of the various compositions are displayed in Equations (3) and (4), respectively. The presented equations provided the best fit; for the equations containing the full set of the acquired factor effects and their interactions, please see the Supplementary Materials. The significant factors and factor interactions are highlighted in boldface in all cases.

y_1_ = 110.11 + **47.02x_1_** + **9.46x_1_^2^** + **19.31x_2_** − **10.04x_3_** − **29.78x_4_** + 2.45x_1_x_2_ + 2.45x_1_^2^x_2_ + 2.29x_1_^2^x_2_^2^ − **6.31x_1_x_3_** − **4.29x_1_^2^x_3_** + **4.96x_1_x_3_^2^** − **3.62x_1_^2^x_3_^2^** − 3.32x_2_x_3_^2^ − **11.48x_2_x_4_** − 2.24x_2_^2^x_4_ − 2.33x_3_x_4_ + **6.83x_3_^2^x_4_** R^2^ = 0.9743 adj. R^2^ = 0.9599 MS Res = 123.02 (3)

y_2_ = 0.133 − **0.053x_1_** − **0.023x_1_^2^** − **0.046x_3_** + **0.018x_4_** + **0.024x_1_x_2_** − **0.019x_1_x_2_^2^** + 0.012x_1_^2^x_3_ + 0.011x_1_^2^x_3_^2^ − 0.014x_1_x_4_ − **0.033x_2_x_3_** − 0.009x_2_x_3_^2^ + 0.010x_2_^2^x_3_^2^ − 0.009x_2_x_4_ + **0.016x_2_^2^x_4_** R^2^ = 0.7627 adj R^2^ = 0.6775 MS Res = 0.0023 (4)

**Figure 1:**
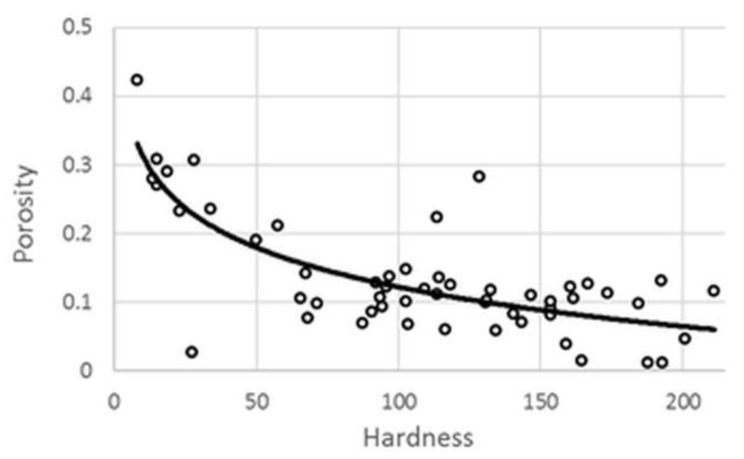
Hardness–porosity plot.

The porosity of the system exhibited a well-established logarithmic correlation with tablet hardness (Figure 1), and the increase in pressure resulted in stronger matrices and decrement in porosity. Nevertheless, there were some outliers, where tablets with low breaking hardness showed extremely low porosity. In such cases, radically increased dwell time was required to avoid critical tableting problems, e.g., capping, lamination, cracking, or picking. This phenomenon caused poorer fitting accuracy in case of porosity (y_2_) values; thus, the results of the statistical analysis and the related conclusions should be treated with caution.

The results revealed that matrices made of EE could reach higher breaking hardness and lower porosity than the tablets made of EL. From the aspect of different weight ratios, the general conclusion is that the composites containing more methacrylate copolymer and less PVC appeared to be the strongest systems with the least porosity, while the 25:75 Eudragit–PVC ratio showed the lowest values and the highest porosity. The mean values showed that paracetamol formed the hardest, and aceclofenac formed the weakest, matrices, but in contrast with the general expectations, this was not directly proportional to the drug release rate, which supports our primary hypothesis that besides the matrix porosity the physicochemical properties of the applied materials, the presence of drug–carrier interactions may considerably influence drug release.

### 3.2. Investigation of Drug–Carrier Interactions

The FT-IR spectra of the compressed samples were used to reveal the presence of the drug–excipient interactions. The method is well applicable in various fields where chemical interactions are the area of interest [23,24]; the present analysis focuses on selected wavenumber ranges reasonably, where signals of H-bond forming groups can be found.

Weak signal intensities were observed in the -OH stretching region (3000–3600 cm^−1^, data not displayed) for all samples, which made the drawing of proper consequences about the interaction status impossible. The further analysis therefore focuses on the C=O stretching (1600–1800 cm^−1^) and the β-OH (β-NH) deformation vibration (1200–1600 cm^−1^) regions.

According to our primary hypothesis, the interaction potential of the studied APIs decreases in the order of ACE > DIS >> PAR, and stronger interactions are expected with EE than with EL in all cases. The results generally confirmed this hypothesis.

**Figure 2:**
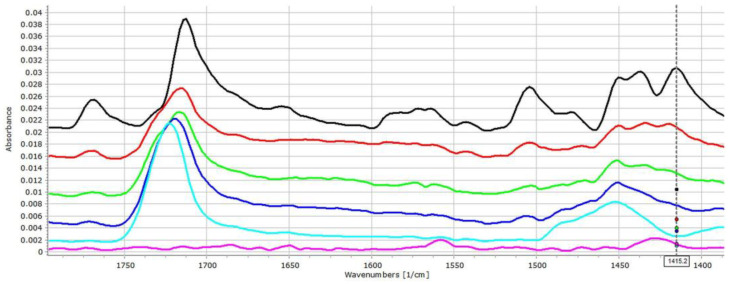
FT-IR spectra (1400–1800 cm^−1^ range) of the ACE (**black**), ACE-PVC:EE 75:25 (**red**), ACE-PVC:EE 50:50 (**green**), ACE-PVC:EE 25:75 (**deep blue**), EE (**light blue**) and PVC (**purple**) samples; multicursor indicates the place of peak shift in the spectrum.

Figure 2 displays the IR spectra of ACE-PVC-EE composition, which exhibits intensive signs of drug–polymer interactions. In the carbonyl signal (C=O stretching) region, EE has a characteristic peak at 1722 cm^−1^, while the ACE at 1712 and at 1769 cm^−1^ belong to the dimerized and monomer forms, respectively. The monomeric peak of ACE (1769 cm^−1^) exhibits decreasing intensity with the increasing amount of EE (Figure 2), indicating strong conjugation between the drug and excipient. Some further changes, such as the shifting of the deformation vibration of the HNC bonds at 1415 cm^−1^ appears to shift to 1420 cm^−1^, which indicates that the corresponding molecular parts may take part in the interaction.

**Figure 3:**
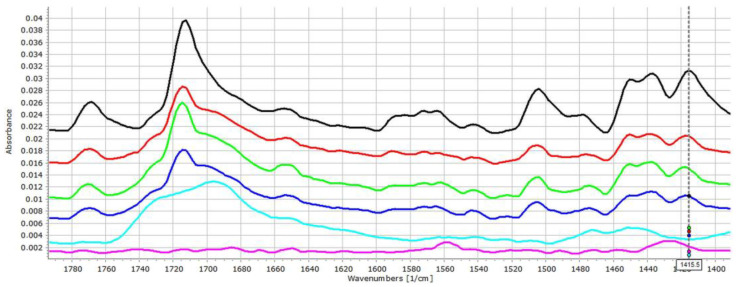
FT-IR spectra (1400–1800 cm^−1^ range) of the ACE (**black**), ACE-PVC:EL 75:25 (**red**), ACE-PVC:EL 50:50 (**green**), ACE-PVC:EL 25:75 (**deep blue**), EL (**light blue**) and PVC (**purple**) samples; multicursor indicates the place of peak shift in the spectrum.

As was expected, ACE exhibited fewer signs of interactions in relation to the acidic EL polymer (Figure 3). The presence of mild interactions by increasing the amount of the EL is supported by the slight shift of the carbonyl signal to 1715, 1717 and 1719 cm^−1^ in the cases of 75:25, 50:50 and 25:75 PVC:EL ratios, respectively, and indicated increasing strength of interactions, which is also supported by the decreasing intensity of the unassociated acidic carbonyl absorption peak which appears at 1769 cm^−1^.

The β-OH vibration of EL appears at 1472.2 cm^−1^, which cannot be seen clearly in the spectra of matrices. A slight shift of the peak at 1415.3 towards 1417.8 cm^−1^ can be recognised, which may reflect some further changes in the environment of the diphenylamine group of the drug. This finding is in accordance with the observation of Liu et al., who confirmed that EL may form H-bond based interactions under proper circumstances [25].

To clarify the importance of the solid-state interactions, and to analyse the effect of the dissolution medium on the strength of interactions, the following experiment was performed. Tablets were dipped into pH 7.4 buffer for 30 min to achieve a complete moisturization of the sample, then the excess of the water was removed, and the samples were measured with FT-IR. Nevertheless, since the presence of water in the pores completely masked the signals in the 3000–3600 and 1550–1700 cm^−1^ regions, the samples were dried in desiccator for 24 h and measured again.

**Figure 4:**
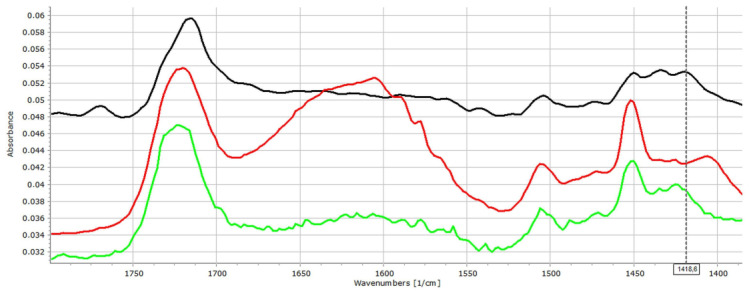
FT-IR spectra of ACE-PVC:EE 75:25 samples: original (**black**), dipped (**red**), and dried (**green**).

Figure 4 displays that the strength of intermolecular interactions has increased as an effect of water absorption. The characteristic peaks of HCN bonds from 1434 cm^−1^ was shifted to 1451 cm^−1^, while the peak at 1420 cm^−1^ shifted to 1405 cm^−1^. The characteristic peak of C-N stretching at 1252 cm^−1^ shifted to 1272 cm^−1^. These changes may also be observed in case of the dried samples, despite some re-organization due to water loss.

**Figure 5:**
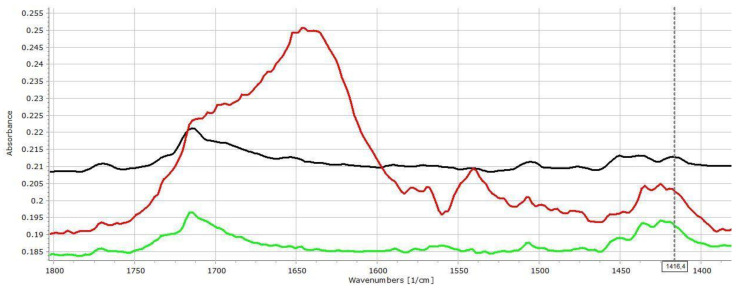
FT-IR spectra of ACE-PVC:EL 75:25 samples: original (**black**), dipped (**red**), and dried (**green**).

In the case of EL containing samples (Figure 5), similar changes may be observed in the characteristic peaks of HCN vibrations: the peak at 1438 cm^−1^ shifts to 1425 cm^−1^ and its intensity increases considerably, while and C-N stretching signal, which may be found at 1279 cm^−1^, shifts to 1291 cm^−1^.

As expected, the matrices prepared with DIS exhibited fewer spectral changes after compression, primarily due the weaker acidity of the drug, but the higher porosity may also play a possible role in this phenomenon. Similarly, as in the ACE-containing compositions, the most obvious changes may be found in the C=O stretching region. The characteristic peak doublet may be found at 1555 and 1571 cm^−1^, where the shift of the peak intensities in the direction of 1571 cm^−1^ may be observed with increasing Eudragit content. As expected, the shift is less intensive in the case of EL-containing compositions (Figure S1) than in EE-containing ones (Figure S2). Some further signs of mild interactions and increased intramolecular association of the C-O bond region of EE-containing matrices may also be observed (Figure S2). The characteristic peak of DIS at 1279 cm^−1^ and of EE at 1269.5 cm^−1^ overlap around 1274 cm^−1^ in accordance with the increasing amount of EE.

The observed interactions were strongly intensified by the dipping. The ratio of the associated C=O groups increased for both EE- and EL-containing matrices (Figure S3 and Figure S4, respectively), which is well visible from the increasing intensity of the associated C=O peak at 1555 cm^−1^ and 1549 cm^−1^ for EE and EL, respectively. Further changes such as the increasing intensity of the peak at 1410 cm^−1^ and a peak shift from 1234 to 1248 cm^−1^, associated with the βNH and C-N stretching vibration, respectively, indicates that under these circumstances, the secondary amino group of DIS was also involved in the association with EE. In the case of EL, the corresponding changes may be found at 1409, 1232, and at 1250 cm^−1^. Other peak shifts from 1168 to 1197 cm^−1^ and from 1190 to 1198 cm^−1^ for EE and EL, respectively, confirm the presence of intermolecular associations from the side of the polymers.

In the case of PAR-containing compositions, they exhibit no obvious signs of interactions in case of EL- (Figure S5) or EE- (Figure S6) containing compositions. The only mentionable change is the slight shift of a C-N stretching peak (PAR) from 1221 to 1224 or 1226 cm^−1^, in the case of EL- and EE-containing compositions, respectively. This finding was in accordance with our primary hypothesis and with the finding of Obediat et al., who also observed no interactions between EE and PAR in compressed matrix systems [26].

Nevertheless, after dipping the tablets into the dissolution medium, considerable changes were observed in the FT-IR spectra for both the EE- and EL-containing samples. The shift of the peaks from 1504 to 1512 cm^−1^ and from 1432 to 1454 cm^−1^ indicates the participation of the secondary amide group, while the shift of the peak from 1224 to 1239 cm^−1^ refers on the involvement of the phenolic -OH group into intermolecular associations (Figure S7). The shift of the characteristic peak of EE from 1170 to 1147 cm^−1^ suggests that the drug is connected to tertiary amino groups of the polymer.

The above-mentioned changes may be observed in case of EL-containing samples (peak shifts from 1504 to 1514 cm^−1^, from 1434 to 1424 cm^−1^ and from 1224 to 1240 cm^−1^), while the shift of the characteristic peak of EL from 1169 to 1186 cm^−1^ indicates that the carbonyl side groups of the polymer are involved into the association (Figure S8).

Overall, the results confirmed that solid-state drug-polymer interactions may be presented after direct compression of the materials, which were in accordance with the findings with de Robertis et al. [11]. The weak solid-state H-bonds may further strengthen during the dissolution process and may influence the drug release rate [12], or in some cases, it may even turn into the formation of polyelectrolyte complexes, as was observed by Pavli et al. [27].

### 3.3. Dissolution Tests and Kinetic Study

The drug release kinetics were evaluated with the use of the Korsmeyer–Peppas model (Table 5), which is the most regular type of dissolution in the case of hydrophilic matrix systems. It can be noticed from the results that the dissolution followed non-Fickian diffusion. Most n values represent non-Fickian kinetics, except for those below 0.45. In the case of cylindrical shapes, Fickian diffusion has a value of 0.45, and for spherical shapes, this value is 0.43. The reason for having smaller values than the limitations of the model is due to the polydisperse nature of the system [28]. Particle size has a major influence on the release exponent. In addition, the geometry of tablets slightly varies from an ideal cylindrical shape.

The PAR-loaded matrices released the most drugs from 40% to 90% within 24 h, the acidic ACE-containing ones released between 2% and 80%, and the DIS-loaded systems released 6–50%. These differences cannot be explained with the different solubilities of the APIs since the applied dissolution environment ensures sink conditions for all tested drugs, and therefore the solubility may not be a limiting factor.

Nevertheless, the results of statistical analysis (Equation (5)) revealed that the main governing forces of the drug dissolution rate (y_3_) are the physicochemical properties, especially the acidic strength of the drug (x_3_) and the applied compression force (x_1_), which confirm that the release rate is primarily determined by the porosity of the tablets, since PAR loaded matrices have the lowest hardness and highest porosity (Table 3.).

y_3_ = 0.937 − **0.380x_1_** − **0.284x_1_^2^** − **0.804x_3_** + 0.185x_4_ − 0.242x_1_x_2_^2^ + **0.403x_1_x_3_** + 0.116x_1_x_3_^2^ + **0.289x_1_^2^x_3_** − 0.208x_1_x_4_ − 0.181x_1_^2^x_4_ − 0.241x_2_x_3_ − 0.135x_2_x_3_^2^ + 0.197x_2_x_4_ + 0.116x_2_^2^x_4_ − **0.440x_3_x_4_** − **0.427x_3_^2^x_4_** R^2^ = 0.7669 adj R^2^ = 0.6661 MS Res = 0.5176 (5)

**Figure 6:**
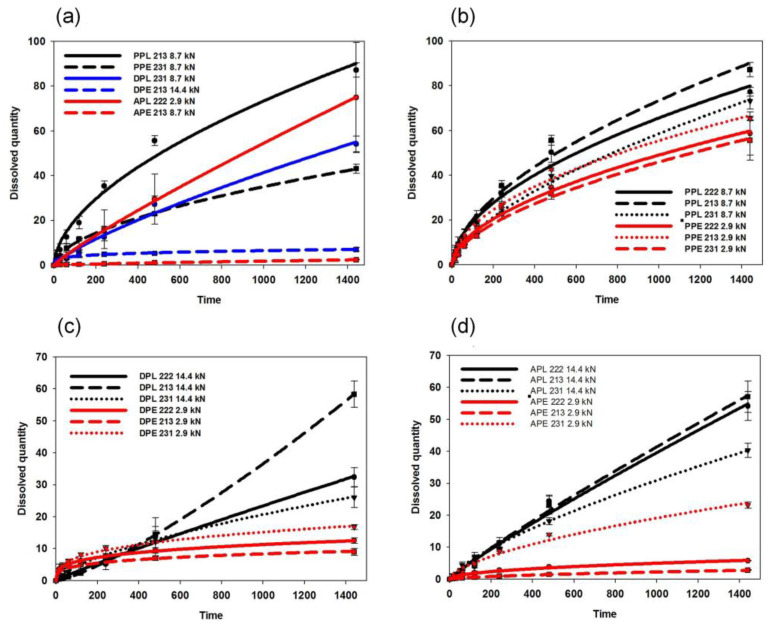
Drug release from various matrices: effect of the drug and polymer properties (**a**) and effect of the composition and compression force for PAR- (**b**), DIS- (**c**) and ACE- (**d**) containing matrices.

The amount of excipient (x_2_), and excipient used (x_4_) exhibited non-significant effects on the dissolution rate, but regarding the significant factor interactions, the compressibility of the API and its possible chemical interactions with the polymer have considerable influence on drug liberation. To confirm this observation and to minimize the effect of the mechanical differences, the dissolution rates of tablets with similar porosities (0.13 ± 0.02) were compared (Figure 6a).

The results met the expectations since EE-based compositions exhibited considerably lower dissolution rates in all cases. Furthermore, in contrast to the observation of Mustafine et al., where in the case of acidic APIs, a dissolution rate of 100% was expected between 2 to 8 h with the application of EE and EL [29], slower dissolution was achieved with all tested systems, which also supports the importance of drug–matrix interactions on the dissolution rate.

The amount of the released drug decreased to 50.6% from 78.5%, to 10.9% from 45.7% and to 6.2% from 58.5% in the cases of PAR-, DIS- and ACE-containing systems, respectively, if EL was switched to EE in the matrix (Figure 6b–d). This may be partially explained by the increased hardness and smaller overall porosity of EE-containing samples, but the tendency is the same if the dissolution rates of samples with similar porosities are compared. In contrast, the observed results are in good accordance with the strength of drug–carrier interactions, since stronger interactions were expected between the acidic drugs and the alkaline EE. This finding supports our previous observation on the role of in situ forming drug–polymer interactions in drug dissolution [12] and were in accordance with the similar findings of Priemel et al. [30] and Mustafine et al. [31].

Finally, to confirm that the interaction-based matrix design is a suitable approach for long-term drug delivery, one-week long dissolution studies were also performed with some selected compositions. The detailed results can be found in the supplementary material.

### 3.4. ANN Modelling

ANN modelling was performed for a dual purpose: to cross check the DoE-based results on the importance of various descriptors on drug dissolution and to compare the effectiveness of kinetic-based and point-to-point approaches in the predictability of the dissolution data. The use of various predictor sets may help the further clarification in which material attributes a play crucial role in the dissolution of the developed implantable matrices.

The results revealed that the overall perfection of point-to-point modelling was significantly (*p* < 0.05) better than kinetic parameter-based models (0.92 ± 0.02 and 0.87 ± 0.03, respectively). No significant difference was observed between the prediction performance of retained networks related to the applied hidden neuron number (Table S3); the results were inconsistent and more dependent on network initialization. Similarly, the use of the various predictor sets caused no considerable change in the overall prediction performance, but an interesting difference was observed between the point-to-point and kinetic parameter-based models when the prediction performance was compared on the train, test, and validation subsets. In the case of kinetic parameter-based modelling, the RMSE of predictions on train (0.22 ± 0.08, 0.15 ± 0.07, 0.09 ± 0.05) and test (0.30 ± 0.04, 0.29 ± 0.05, 0.19 ± 0.05) datasets were significantly improved in order of Approach 1, 2 and 3, respectively, while no significant change was observed in the validation dataset. In contrast, for point-to-point modelling, the RMSE of predictions on train and test datasets remained unchanged, while it was a significant improvement on the validation dataset (328 ± 19, 235 ± 18, 158 ± 10) in order of approaches 5, 4, and 6, respectively.

Nevertheless, despite that the observed differences can be stated, the use of continuous inputs with appropriate descriptors of the tablet texture (hardness, porosity) enabled for the best prediction performance for both point-to-point and kinetic parameter-based approaches, despite the fact that the texture parameters exhibited relatively low importance in predictivity according to the results of the global sensitivity analysis (Table S4).

The results of the global sensitivity analysis, which show the relative importance of various predictors (inputs) on prediction outcome, are partly consistent with the results of the experimental design. The greatest effect was observed for drug solubility, but the pKa of the excipients and the peak shift indicating drug–excipient interactions exhibited similar importance versus the compression pressure, tablet texture or the amount of excipient (Table S4).

**Figure 7:**
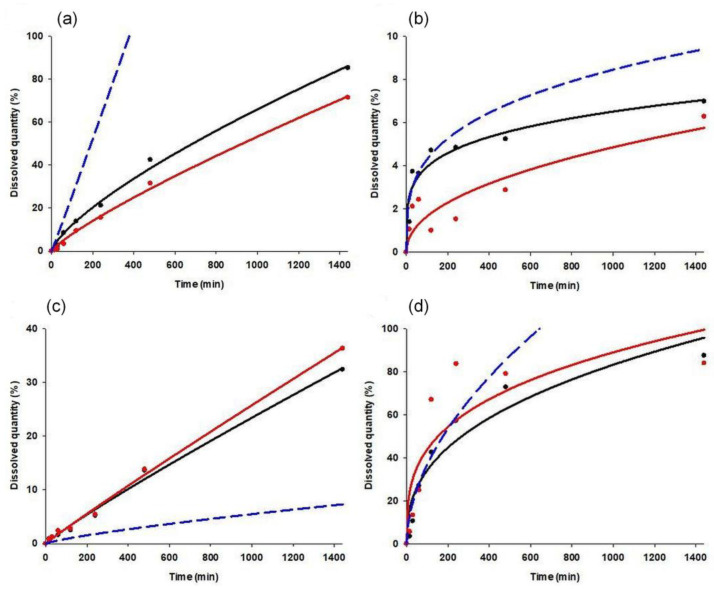
Observed and predicted dissolution curves of case 43 (**a**), case 36 (**b**), case 24 (**c**) and case 7 (**d**). Black dots: observed data points; black line: fitted model on observed data; red dots: predicted data points; red line: fitted model on predicted data points; blue line: predicted model.

To ultimately compare the prediction effectiveness of point-to-point and kinetic parameter-based modelling approaches, the best performing networks (best overall training perfection and lowest prediction error on validation dataset, with no negative prediction values) were selected for both modelling approaches. The best network for kinetic parameter-based modelling had nine input, seven hidden and two output neurons, with logistic activation on hidden and exponential on output neurons (training perfection: 0.9103, validation error: 0.0760). The structure of the best point-to-point network was nine input, sixteen hidden, and seven output neurons, with exponential activation on hidden neurons and logistic on output neurons (training perfection: 0.9286, validation error: 83.06). Figure 7 shows the predicted dissolution curves of the best and worst predicted cases.

It is clearly visible that the point-to-point modelling approach showed more consistent accuracy, especially in cases where drug release was nearly linear (Figure 7a,c). In other cases, the most considerable inaccuracies were observed at the 2, 4, and 8 h data points. Nevertheless, the predicted results were closer to the observed ones as for kinetic parameter-based models.

The main problem of the kinetic parameter-based approaches is that the dissolution data obtained show large differences; thus, the corresponding dissolution rates were scattered by two orders of magnitude. This posed a certain limitation on the predictivity since small inaccuracies in the predicted values of low release rates resulted in predictions of 3-5-fold faster or slower dissolution (Figure 7a,c). The prediction of release exponents, which scattered in a significantly smaller range, was more accurate, but the presence of small inaccuracies can also cause large differences between the observed and predicted data at the end of the dissolution curves. It can be stated that the obtained models provide the desired accuracy only in the first 4–8 h of dissolution (Figure 7b,d).

## 4. Conclusions

The present study introduced a comprehensive systematic approach for the investigation of the role of chemical interactions between APIs and excipients in directly compressed matrices. With the help of FT-IR studies, it was confirmed that solid-state interactions may be induced in directly compressed matrix systems. The results also confirmed that the presence of solid-state interactions may not always present in directly compressed systems, but their presence will definitely predict strong in situ forming interactions during the drug dissolution process. The interactions are mostly H-bond based, but the forming of polyelectrolyte complexes cannot be excluded. The results of both DoE analysis and the ANN modelling supported our primary hypothesis that API-excipient interactions have a considerable effect on drug release by retaining the drug in the matrix.

The secondary hypothesis that kinetic parameters can be effectively used as an output in predicting drug release during ANN modelling has not been substantiated. The simplified structure did not result in a faster generalization of the network, and due to the wide scatter of the output results, small variations in the predicted release rate caused a high degree of uncertainty in the predicted dissolution curves. In contrast, when a point-to-point approach is used, the difference between the observed and predicted data at a given time can be compensated for at other time points. Therefore, applying a point-to-point approach provides greater reliability and more reliable predictions.

Nevertheless, the above-mentioned limitations can be overcome by increasing the number of cases, which makes it possible to fill in the gaps in the training data set. Therefore, the combination of interaction factors and ANN-based modelling may be a promising way to design extended-release products, especially implantable systems with tailored dissolution properties.

## Figures and Tables

**Table 1:** Physicochemical properties of raw materials.

	Paracetamol	Diclofenac Sodium	Aceclofenac	EUDR-E	EUDR-L	PVC
Solubility (pH 7.4)	18–21 mg/mL	5.15 mg/mL	4.0–12.8 mg/mL	<10 µg/mL (>pH 5)	<0.03 mg/mL (>pH 6)	<10 µg/mL
logP	0.34	3.10	4.16	-	-	-
pKa	9.46	4.00	3.44	10	6	-
H+ acceptor	2	2	4	3/n*	3/n *	-
H+ donor	2	2	2	0/n*	1/n *	-
Rotable bonds	3	3	5	-	-	-

* x/n: represents the number of H+ acceptor/donor groups per monomer.

**Table 2:** DoE plan.

Compression Pressure (MPa) (x_1_)		Eudragit/PVC Ratio (%/%) (x_2_)		API (x_3_)		Eudragit Type (x_4_)	
Level	Value	Level	Value	Level	Value	Level	Value
−1	75	−1	25/75	−1	Paracetamol	−1	Eudragit E
						+1	Eudragit L
				0	Diclofenac Sodium	−1	Eudragit E
						+1	Eudragit L
				+1	Aceclofenac	−1	Eudragit E
						+1	Eudragit L
		0	50/50	−1	Paracetamol	−1	Eudragit E
						+1	Eudragit L
				0	Diclofenac Sodium	−1	Eudragit E
						+1	Eudragit L
				+1	Aceclofenac	−1	Eudragit E
						+1	Eudragit L
		+1	75/25	−1	Paracetamol	−1	Eudragit E
						+1	Eudragit L
				0	Diclofenac Sodium	−1	Eudragit E
						+1	Eudragit L
				+1	Aceclofenac	−1	Eudragit E
						+1	Eudragit L
0	225	−1	25/75	−1	Paracetamol	−1	Eudragit E
						+1	Eudragit L
				0	Diclofenac Sodium	−1	Eudragit E
						+1	Eudragit L
				+1	Aceclofenac	−1	Eudragit E
						+1	Eudragit L
		0	50/50	−1	Paracetamol	−1	Eudragit E
						+1	Eudragit L
				0	Diclofenac Sodium	−1	Eudragit E
						+1	Eudragit L
				+1	Aceclofenac	−1	Eudragit E
						+1	Eudragit L
		+1	75/25	−1	Paracetamol	−1	Eudragit E
						+1	Eudragit L
				0	Diclofenac Sodium	−1	Eudragit E
						+1	Eudragit L
				+1	Aceclofenac	−1	Eudragit E
						+1	Eudragit L
+1	375	−1	25/75	−1	Paracetamol	−1	Eudragit E
						+1	Eudragit L
				0	Diclofenac Sodium	−1	Eudragit E
						+1	Eudragit L
				+1	Aceclofenac	−1	Eudragit E
						+1	Eudragit L
		0	50/50	−1	Paracetamol	−1	Eudragit E
						+1	Eudragit L
				0	Diclofenac Sodium	−1	Eudragit E
						+1	Eudragit L
				+1	Aceclofenac	−1	Eudragit E
						+1	Eudragit L
		+1	75/25	−1	Paracetamol	−1	Eudragit E
						+1	Eudragit L
				0	Diclofenac Sodium	−1	Eudragit E
						+1	Eudragit L
				+1	Aceclofenac	−1	Eudragit E
						+1	Eudragit L

**Table 3:** Input variables of the various ANN training approaches.

Modelling Type	Approach 1	Approach 2	Approach 3	Approach 4	Approach 5	Approach 6
	Kinetic Based	Kinetic Based	Kinetic Based	Point-to-Point	Point-to-Point	Point-to-Point
Input variable						
Drug	x			x		
Drug solubility (mg/mL)		x	x		x	x
Drug pKa		x	x		x	x
Excipient	x			x		
Excipient solubility (mg/mL)		x	x		x	x
Excipient pKa		x	x		x	x
Excipient amount (%)	x	x	x	x	x	x
Compression pressure (MPa)	x	x	x	x	x	x
Hardness			x			x
Porosity			x			x
Peak Shift	x	x	x	x	x	x

**Table 4:** Physical parameters of matrices.

Eudragit/PVC Ratio (%/%)	Compression Pressure (MPa)	Composition	Mass (mg)	Hardness (N)	Poro-Sity	Composition	Mass (mg)	Hardness (N)	Porosity	Composition	Mass (mg)	Hardness (N)	Porosity
25/75	75	PAR-PVC-EL	147.0	15.0	0.307	DIS-PVC-EL	152.1	8.0	0.423	ACE-PVC-EL	144.5	13.5	0.279
	225		147.9	114.3	0.136		135.2	118.5	0.125		131.4	68.2	0.077
	375		141.6	109.1	0.118		202.6	140.7	0.082		131.2	103.6	0.067
50/50	75		149.0	18.7	0.290		154.2	34.0	0.236		152.7	22.9	0.232
	225		135.9	95.8	0.122		138.7	92.0	0.128		137.8	65.8	0.104
	375		142.2	133.6	0.111		146.9	134.4	0.058		142.4	71.3	0.097
75/25	75		147.0	28.2	0.306		153.8	27.3	0.026		147.7	15.2	0.027
	225		151.7	132.6	0.118		136.9	97.0	0.137		134.9	90.7	0.086
	375		149.7	153.8	0.091		121.5	153.8	0.100		137.1	116.7	0.060
25/75	75	PAR-PVC-EE	154.8	113.6	0.223	DIS-PVC-EE	150.4	94.4	0.092	ACE-PVC-EE	147.3	93.4	0.106
	225		142.5	166.6	0.126		129.0	130.7	0.098		151.2	159.1	0.038
	375		136.1	184.5	0.098		130.7	173.7	0.113		152.7	164.7	0.014
50/50	75		149.0	50.1	0.191		151.7	57.4	0.210		151.1	67.6	0.141
	225		152.2	146.7	0.109		138.8	102.6	0.101		137.6	87.5	0.068
	375		145.2	161.8	0.105		138.7	153.8	0.080		139.0	143.6	0.071
75/25	75		149.5	128.6	0.282		137.4	102.9	0.147		146.3	131.2	0.102
	225		137.0	192.4	0.131		135.8	160.6	0.121		145.9	187.9	0.011
	375		148.6	211.2	0.115		151.7	201.0	0.046		141.7	192.8	0.011

**Table 5:** Release rates and release exponents derived from the Korsmeyer–Peppas model (24 h long test).

Eudragit/PVC Ratio (%/%)	Compression Pressure (MPa)	Composition	R^2^	k	n	Composition	R^2^	k	n	Composition	R^2^	k	n
25/75	75	PAR-PVC-EL	0.9230	5.7937	0.3858	DIS-PVC-EL	0.9966	0.0419	0.9833	ACE-PVC-EL	0.9945	0.4184	0.7325
	225		0.9840	1.4970	0.5635		0.9997	0.0092	1.1609		0.9979	0.0540	0.9340
	375		0.9870	1.7224	0.5345		0.9999	0.0054	1.2765		0.9976	0.0850	0.8940
50/50	75		0.9410	6.8732	0.3534		0.9867	1.4394	0.4317		0.9994	0.1289	0.8756
	225		0.9845	1.6004	0.5377		0.9979	0.0265	0.9560		0.9691	0.1877	0.8271
	375		0.9712	1.5522	0.5594		0.9942	0.0435	0.9105		0.9899	0.0829	0.8931
75/25	75		0.9920	1.7155	0.5058		0.9789	1.5287	0.5567		0.9906	0.2466	0.7311
	225		0.9974	0.7570	0.6298		0.9902	0.1828	0.7850		0.9990	0.2002	0.7389
	375		0.9928	1.5108	0.5239		0.9976	0.2207	0.6573		0.9991	0.2055	0.7260
25/75	75	PAR-PVC-EE	0.9936	0.9010	0.5693	DIS-PVC-EE	0.9660	1.3715	0.2623	ACE-PVC-EE	0.9998	0.0376	0.5936
	225		0.9977	0.6775	0.5774		0.9609	1.4599	0.2306		0.9933	0.0097	0.7596
	375		0.9755	0.8078	0.5483		0.9286	1.4502	0.2176		0.9820	0.0106	0.7241
50/50	75		0.9915	1.1441	0.5441		0.9974	1.7063	0.2739		0.9877	0.2063	0.4619
	225		0.9973	0.4156	0.6751		0.9846	0.9046	0.3542		0.9972	0.0704	0.5474
	375		0.9978	0.3755	0.6785		0.9823	0.9284	0.3404		0.9969	0.0805	0.4910
75/25	75		0.9920	1.6898	0.5056		0.9867	1.5953	0.3265		0.9839	0.2829	0.6100
	225		0.9983	0.7675	0.5535		0.9659	1.0545	0.3185		0.9933	0.1695	0.5134
	375		0.9918	0.8430	0.5239		0.9641	1.5575	0.2903		0.9670	0.2069	0.4661

## Data Availability

The detailed dataset of ANN predictions in this study are available on request from the corresponding author due size and file format.

## Supplementary Materials

The following supporting information can be downloaded at: https://www.mdpi.com/article/10.3390/pharmaceutics14020228/s1, Table S1: Dataset of the ANN modelling; Figure S1 FT-IR spectra (650-1800 cm^-1^ range) of the DIS (black); DIS-PVC:EL 75:25 (red); DIS-PVC:EL 50:50 (green); DIS-PVC:EL 25:75 (deep blue); EL (light blue) and PVC (purple) samples; Figure S2. FT-IR spectra (650-1800 cm^-1^ range) of the DIS (black); DIS-PVC:EE 75:25 (red); DIS-PVC:EE 50:50 (green); DIS-PVC:EE 25:75 (deep blue); EE (light blue) and PVC (purple) samples; Figure S3. FT-IR spectra of Dis-PVC:EE 75:25 samples: original (black), dipped (red), and dried (green); Figure S4. FT-IR spectra of Dis-PVC:EL 75:25 samples: original (black), dipped (red), and dried (green); Figure S5: FT-IR spectra (650-1800 cm^-1^ range) of the PAR (black); PAR-PVC:EL 75:25 (red); PAR-PVC:EL 50:50 (green); PAR-PVC:EL 25:75 (deep blue); EL (light blue) and PVC (purple) samples; Figure S6: FT-IR spectra (650-1800 cm^-1^ range) of the PAR (black); PAR-PVC:EE 75:25 (red); PAR-PVC:EE 50:50 (green); PAR-PVC:EE 25:75 (deep blue); EE (light blue) and PVC (purple) samples; Figure S7. FT-IR spectra of Par-PVC:EE 75:25 samples: original (black), dipped (red), and dried (green); Figure S8. FT-IR spectra of Par-PVC:EL 75:25 samples: original (black), dipped (red), and dried (green); Table S2. Release rates and release exponents derived from the Korsmeyer-Peppas model (1-week-long test); Figure S9. Drug release from various matrices: effect of the drug and polymer properties (a) and effect of the composition and compression force for PAR (b), DIS (c) and ACE (d) containing matrices; Table S3: Performance of the neurons; Table S4: Results of the global sensitivity analysis.
