# Supplementary material for: Predicting Drug Release Rate of Implantable Matrices and Better Understanding of the Underlying Mechanisms through Experimental Design and Artificial Neural Network-Based Modelling
Source: Pharmaceutics. 2022 Jan 19;14(2):228. doi: 10.3390/pharmaceutics14020228 (PMC8879093; doi:10.3390/pharmaceutics14020228)
Supplement: Supplementary file 1 [file pharmaceutics-14-00228-s001.zip › pharmaceutics-1515122-supplementary.pdf]

pharmaceutics MDPI

Supplementary Material: Predicting Drug Release Rate of Implantable Matrices and Better Understanding of the Underlying Mechanisms through Experimental Design and Artificial Neural Network-Based Modelling

Ernő Benkő, Ilija German Ilič, Katalin Kristó, Géza Regdon Jr., Ildikó Csóka, Klára Pintye-Hódi, Stane Srčič and Tamás Sovány *

Table S1. Dataset of the ANN modelling.

	Subset	Drug	Drug solubility (mg/ml)	Drug pKa	Exci.	Exc. amount*	Excipient solubility (mg/ml)	Excipient pKa	Compression pressure (MPa)	Hardness (N)	Porosity	Peak shift	Release rate	Release exponent	15 min	30 min	60 min	120 min	240 min	480 min	1440 min
1	Train	Par.	19,5	9,46	Eudragit L	22	0,03	4,6	75	15	0,307	3	5,79	0,39	6,06	9,63	12,81	16,80	27,32	43,19	66,47
2	Validation	Par.	19,5	9,46	Eudragit L	22	0,03	4,6	225	114,3	0,136	3	1,50	0,56	3,20	6,58	9,23	14,68	22,88	39,68	73,09
3	Test	Par.	19,5	9,46	Eudragit L	22	0,03	4,6	375	109,1	0,118	3	1,72	0,53	4,71	6,91	10,14	19,76	29,50	39,30	67,22
4	Train	Par.	19,5	9,46	Eudragit L	33	0,03	4,6	75	18,7	0,29	6	6,87	0,35	7,31	12,17	21,78	41,80	63,28	72,20	79,28
5	Test	Par.	19,5	9,46	Eudragit L	33	0,03	4,6	225	95,8	0,122	6	1,60	0,54	4,52	6,22	10,77	21,65	31,56	50,28	77,27
6	Train	Par.	19,5	9,46	Eudragit L	33	0,03	4,6	375	133,6	0,111	6	1,55	0,56	3,34	6,98	10,17	18,91	36,13	59,22	86,65
7	Validation	Par.	19,5	9,46	Eudragit L	44	0,03	4,6	75	28,2	0,306	13	1,72	0,51	3,32	10,55	26,95	42,46	57,16	72,80	87,48
8	Validation	Par.	19,5	9,46	Eudragit L	44	0,03	4,6	225	132,6	0,118	13	0,76	0,63	3,82	6,86	12,57	18,93	35,31	55,68	87,21
9	Train	Par.	19,5	9,46	Eudragit L	44	0,03	4,6	375	153,8	0,091	13	1,51	0,52	5,37	8,14	10,77	21,18	36,54	50,66	81,78
10	Train	Par.	19,5	9,46	Eudragit E	22	0,01	10	75	113,6	0,223	9	0,90	0,57	5,95	9,48	12,63	16,52	26,86	42,48	65,35
11	Validation	Par.	19,5	9,46	Eudragit E	22	0,01	10	225	166,6	0,126	9	0,68	0,58	3,98	3,97	7,68	11,63	15,86	22,90	43,10
12	Train	Par.	19,5	9,46	Eudragit E	22	0,01	10	375	184,5	0,098	9	0,81	0,55	5,85	5,50	7,78	9,38	14,29	20,56	38,63
13	Train	Par.	19,5	9,46	Eudragit E	33	0,01	10	75	50,1	0,191	11	1,14	0,54	2,97	5,52	9,13	14,52	25,64	34,54	58,69
14	Test	Par.	19,5	9,46	Eudragit E	33	0,01	10	225	146,7	0,109	11	0,42	0,68	1,43	4,10	6,74	9,30	16,41	28,71	55,84
15	Train	Par.	19,5	9,46	Eudragit E	33	0,01	10	375	161,8	0,105	11	0,38	0,68	4,15	3,65	5,70	8,61	15,81	24,97	52,17
16	Test	Par.	19,5	9,46	Eudragit E	44	0,01	10	75	128,6	0,282	11	1,69	0,51	2,74	4,38	8,30	12,80	22,73	31,77	55,66
17	Train	Par.	19,5	9,46	Eudragit E	44	0,01	10	225	192,4	0,131	11	0,77	0,55	1,70	4,53	7,21	10,55	17,07	24,09	44,89
18	Train	Par.	19,5	9,46	Eudragit E	44	0,01	10	375	211,2	0,115	11	0,84	0,52	1,66	3,52	6,33	9,59	16,77	28,68	41,73
19	Validation	Dic.	5,15	4	Eudragit L	22	0,03	4,6	75	8	0,423	17	0,04	0,98	2,30	3,95	12,95	23,54	32,46	54,95	84,53
20	Test	Dic.	5,15	4	Eudragit L	22	0,03	4,6	225	118,5	0,125	17	0,01	1,16	0,77	1,37	2,87	6,16	12,53	27,13	54,17

21	Train	Dic.	5,15	4	Eudragit L	22	0,03	4,6	375	140,7	0,082	17	0,01	1,28	1,30	1,62	2,68	5,12	7,98	13,59	26,05
22	Train	Dic.	5,15	4	Eudragit L	33	0,03	4,6	75	34	0,236	17	1,44	0,43	3,79	4,63	10,60	11,68	14,13	21,69	32,92
23	Train	Dic.	5,15	4	Eudragit L	33	0,03	4,6	225	92	0,128	17	0,03	0,96	1,11	1,18	1,34	2,49	4,38	10,00	27,66
24	Validation	Dic.	5,15	4	Eudragit L	33	0,03	4,6	375	134,4	0,058	17	0,04	0,91	0,86	1,14	1,59	2,52	5,18	13,60	32,40
25	Train	Dic.	5,15	4	Eudragit L	44	0,03	4,6	75	27,3	0,026	17	1,53	0,56	0,44	0,82	1,96	3,86	7,52	20,22	53,14
26	Train	Dic.	5,15	4	Eudragit L	44	0,03	4,6	225	97	0,137	17	0,18	0,79	0,42	0,58	1,19	2,47	4,80	12,17	42,56
27	Train	Dic.	5,15	4	Eudragit L	44	0,03	4,6	375	153,8	0,1	17	0,22	0,66	0,50	0,60	1,21	2,38	5,59	14,51	58,30
28	Validation	Dic.	5,15	4	Eudragit E	22	0,01	10	75	94,4	0,092	14	1,37	0,26	2,52	4,70	6,25	8,20	10,13	12,14	16,73
29	Test	Dic.	5,15	4	Eudragit E	22	0,01	10	225	130,7	0,098	14	1,46	0,23	1,18	2,95	4,42	5,67	6,08	7,56	10,45
30	Train	Dic.	5,15	4	Eudragit E	22	0,01	10	375	173,7	0,113	14	1,45	0,22	1,76	4,05	5,44	7,09	8,19	9,55	12,33
31	Train	Dic.	5,15	4	Eudragit E	33	0,01	10	75	57,4	0,21	12	1,71	0,27	3,59	4,45	5,44	6,23	7,24	9,43	12,55
32	Train	Dic.	5,15	4	Eudragit E	33	0,01	10	225	102,6	0,101	12	0,90	0,35	1,37	3,15	4,16	4,92	6,35	8,62	11,55
33	Train	Dic.	5,15	4	Eudragit E	33	0,01	10	375	153,8	0,08	12	0,93	0,34	1,48	2,53	4,24	5,11	6,06	7,94	10,72
34	Test	Dic.	5,15	4	Eudragit E	44	0,01	10	75	102,9	0,147	9	1,60	0,33	1,57	3,55	4,47	5,10	6,07	7,01	8,94
35	Test	Dic.	5,15	4	Eudragit E	44	0,01	10	225	160,6	0,121	9	1,05	0,32	1,70	3,86	4,05	4,50	5,18	6,03	7,72
36	Validation	Dic.	5,15	4	Eudragit E	44	0,01	10	375	201	0,046	9	1,56	0,29	1,40	3,74	3,64	4,72	4,85	5,25	6,99
37	Train	Acec.	8,4	3,44	Eudragit L	22	0,03	4,6	75	13,5	0,279	41	0,42	0,73	1,00	1,26	3,04	6,70	13,98	25,53	49,32
38	Train	Acec.	8,4	3,44	Eudragit L	22	0,03	4,6	225	68,2	0,077	41	0,05	0,93	0,68	1,80	3,79	7,03	11,98	19,34	43,03
39	Test	Acec.	8,4	3,44	Eudragit L	22	0,03	4,6	375	103,6	0,067	41	0,09	0,89	1,02	1,63	4,38	7,10	10,97	18,10	40,34
40	Train	Acec.	8,4	3,44	Eudragit L	33	0,03	4,6	75	22,9	0,232	41	0,13	0,88	1,72	2,20	3,71	7,58	16,05	29,45	74,91
41	Train	Acec.	8,4	3,44	Eudragit L	33	0,03	4,6	225	65,8	0,104	41	0,19	0,83	0,70	1,39	2,87	6,06	12,11	40,87	74,86
42	Validation	Acec.	8,4	3,44	Eudragit L	33	0,03	4,6	375	71,3	0,097	41	0,08	0,89	0,37	1,12	1,56	4,01	9,36	24,38	54,10
43	Validation	Acec.	8,4	3,44	Eudragit L	44	0,03	4,6	75	15,2	0,027	41	0,25	0,73	0,51	2,58	8,49	13,88	21,22	42,42	85,16
44	Train	Acec.	8,4	3,44	Eudragit L	44	0,03	4,6	225	90,7	0,086	41	0,20	0,74	0,19	0,53	1,37	3,86	9,33	18,27	47,89
45	Train	Acec.	8,4	3,44	Eudragit L	44	0,03	4,6	375	116,7	0,06	41	0,21	0,73	0,30	0,83	2,21	5,24	11,42	23,10	57,08
46	Validation	Acec.	8,4	3,44	Eudragit E	22	0,01	10	75	93,4	0,106	46	0,04	0,59	0,90	1,20	2,21	4,65	8,76	13,97	23,18
47	Train	Acec.	8,4	3,44	Eudragit E	22	0,01	10	225	159,1	0,038	46	0,01	0,76	0,55	1,22	1,60	2,09	2,66	3,78	7,21
48	Test	Acec.	8,4	3,44	Eudragit E	22	0,01	10	375	164,7	0,014	46	0,01	0,72	2,66	1,49	1,84	1,82	2,24	3,34	6,37
49	Test	Acec.	8,4	3,44	Eudragit E	33	0,01	10	75	67,6	0,141	47	0,21	0,46	0,48	0,70	1,23	1,99	2,80	3,85	5,76
50	Train	Acec.	8,4	3,44	Eudragit E	33	0,01	10	225	87,5	0,068	47	0,07	0,55	0,20	0,46	0,66	0,90	1,43	2,18	3,73

5 1	Validation	Acec.	8,4	3,44	Eudragit E	33	0,01	10	375	143,6	0,071	47	0,08	0,49	0,32	0,52	0,65	0,79	1,13	1,65	2,89
5 2	Test	Acec.	8,4	3,44	Eudragit E	44	0,01	10	75	131,2	0,102	49	0,28	0,61	0,20	0,27	0,42	0,64	0,96	1,49	2,81
5 3	Train	Acec.	8,4	3,44	Eudragit E	44	0,01	10	225	187,9	0,011	49	0,17	0,51	0,22	0,20	0,24	0,33	0,57	1,05	2,43
5 4	Train	Acec.	8,4	3,44	Eudragit E	44	0,01	10	375	192,8	0,011	49	0,21	0,47	0,26	0,21	0,24	0,32	0,44	0,93	2,06

*22, 33 and 44 % corresponds with 25:75, 50:50 and 75:25 Eudragit:PVC ratio, respectively.

Full equations:

$$y_1=110.12+\mathbf{47.05x_1}+\mathbf{9.44x_1^2}+\mathbf{19.34x_2}-0.44x_2^2-\mathbf{10.12x_3}+0.36x_3^2-\mathbf{29.48x_4}+2.50x_1x_2-1.64x_1x_2^2+2.42x_1^2x_2+2.31x_1^2x_2^2-\mathbf{6.44x_1x_3}+\mathbf{4.80x_1x_3^2}-4.22x_1^2x_3-3.54x_1^2x_3^2+\mathbf{4.54x_1x_4}+3.20x_1^2x_4+1.33x_2x_3-3.47x_2x_3^2+1.30x_2^2x_3+0.56x_2^2x_3^2-\mathbf{11.48x_2x_4}-2.23x_2^2x_4-2.32x_3x_4+\mathbf{6.83x_3^2x_4} \quad \text{(S1)}$$

$R^2=0.9756$ adj $R^2=0.9537$ MS Residual: 141.79

$$y_2=0.132-\mathbf{0.054x_1}-\mathbf{0.022x_1^2}-0.009x_2+0.006x_2^2-\mathbf{0.043x_3}-0.0003x_3^2+\mathbf{0.018x_4}+0.022x_1x_2-0.018x_1x_2^2+0.004x_1^2x^2-0.006x_1^2x_2^2+0.009x_1x_3+0.002x_1x_3^2+0.010x_1^2x_3+0.009x_1^2x_3^2-0.014x_1x_4-0.004x_1^2x_4-\mathbf{0.029x_2x_3}-0.006x_2x_3^2+0.006x_2^2x_3+0.009x_2^2x_3^2-0.009x_2x_4+\mathbf{0.016x_2^2x_4}+0.007x_3x_4-0.0006x_3^2x_4 \quad \text{(S2)}$$

$R^2=0.7928$ adj $R^2=0.6079$ MS Res=0.0028

$$y_3=0.936-\mathbf{0.381x_1}-\mathbf{0.283x_1^2}+0.092x_2+0.038x_2^2-\mathbf{0.804x_3}-0.026x_3^2+0.185x_4-0.011x_1x_2-0.241x_1x_2^2-0.033x_1^2x_2-0.042x_1^2x_2^2+\mathbf{0.403x_1x_3}+0.113x_1x_3^2+0.289x_1^2x3+0.046x_1^2x_3^2-0.208x_1x_4-0.181x_1^2x_4-0.242x_2x_3-0.137x_2x_3^2-0.097x_2^2x_3-0.063x_2^2x_3^2+0.197x_2x_4+0.116x_2^2x_4-\mathbf{0.440x_3x_4}-\mathbf{0.427x_3^2x^4} \quad \text{(S3)}$$

R2=0.7801 adj R2=0.5838 MS Res=0.6453

Supplementary Figures for FT-IR analysis:

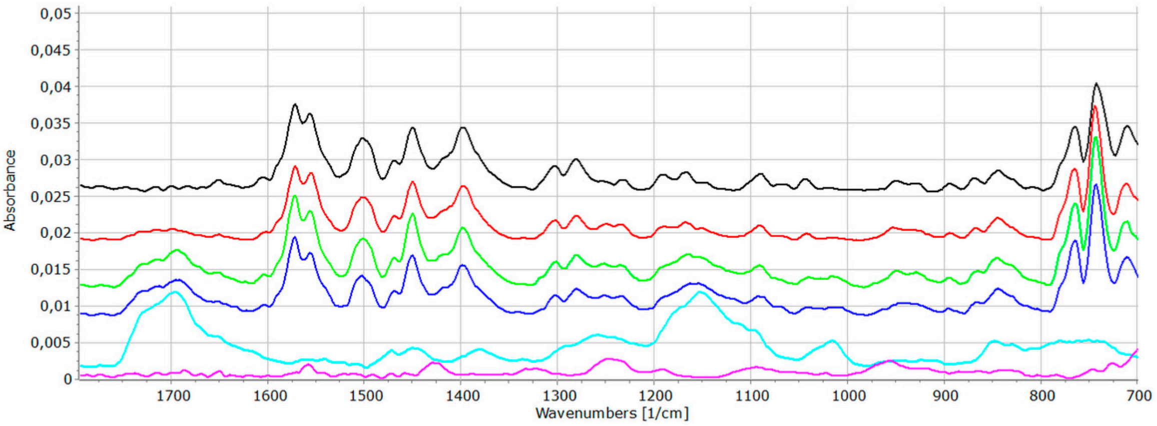

Figure S1. FT-IR spectra (650-1800 cm^{-1} range) of the DIS (black); DIS-PVC:EL 75:25 (red); DIS-PVC:EL 50:50 (green); DIS-PVC:EL 25:75 (deep blue); EL (light blue) and PVC (purple) samples.

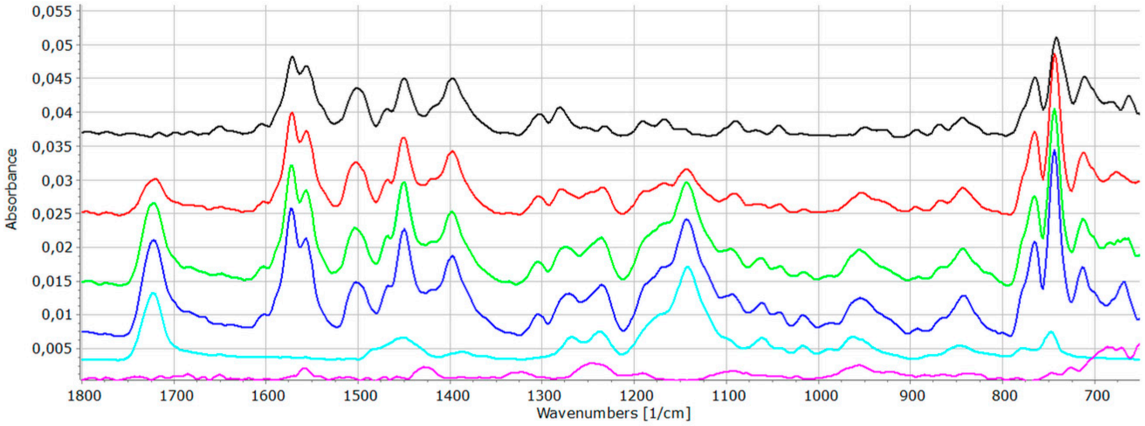

Figure S2. FT-IR spectra (650-1800 cm^{-1} range) of the DIS (black); DIS-PVC:EE 75:25 (red); DIS-PVC:EE 50:50 (green); DIS-PVC:EE 25:75 (deep blue); EE (light blue) and PVC (purple) samples.

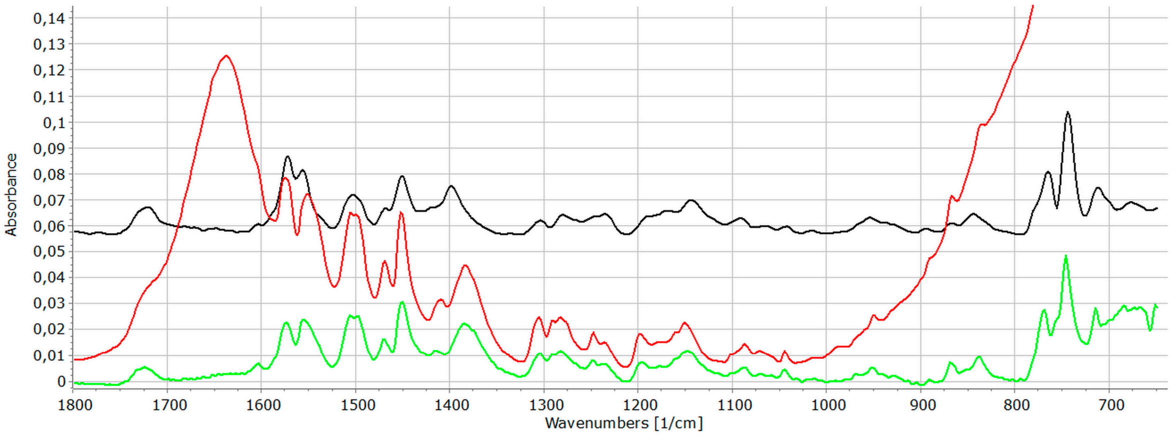

Figure S3. FT-IR spectra of Dis-PVC:EE 75:25 samples: original (black), dipped (red), and dried (green).

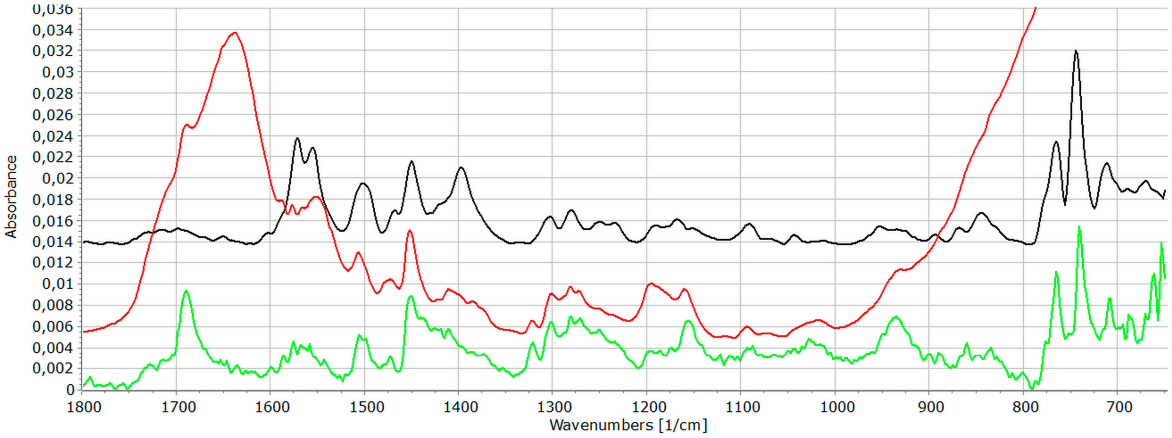

Figure S4. FT-IR spectra of Dis-PVC:EL 75:25 samples: original (black), dipped (red), and dried (green).

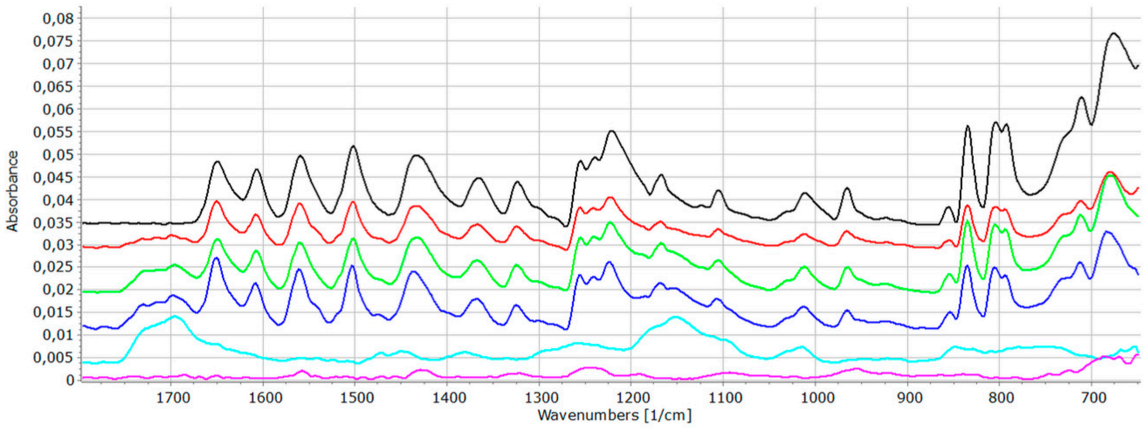

Figure S5. FT-IR spectra (650-1800 cm^{-1} range) of the PAR (black); PAR-PVC:EL 75:25 (red); PAR-PVC:EL 50:50 (green); PAR-PVC:EL 25:75 (deep blue); EL (light blue) and PVC (purple) samples.

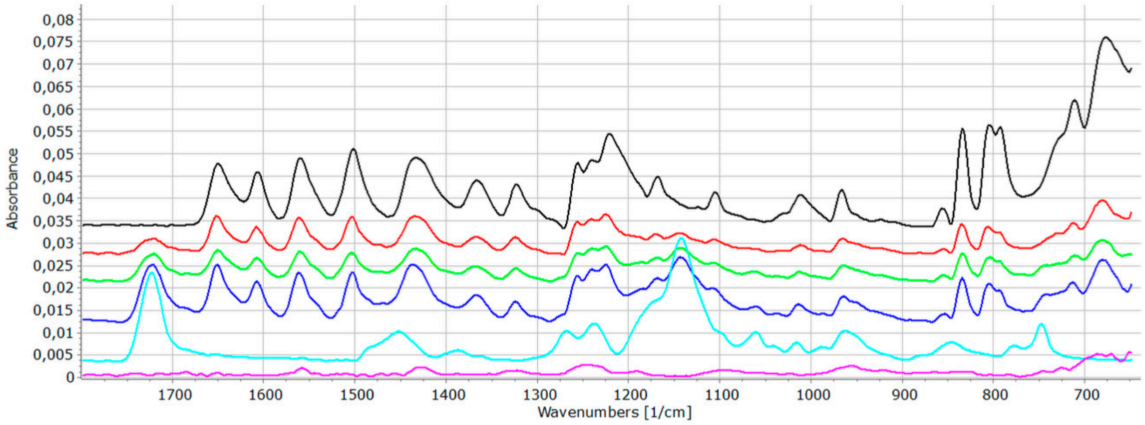

Figure S6. FT-IR spectra (650-1800 cm^{-1} range) of the PAR (black); PAR-PVC:EE 75:25 (red); PAR-PVC:EE 50:50 (green); PAR-PVC:EE 25:75 (deep blue); EE (light blue) and PVC (purple) samples.

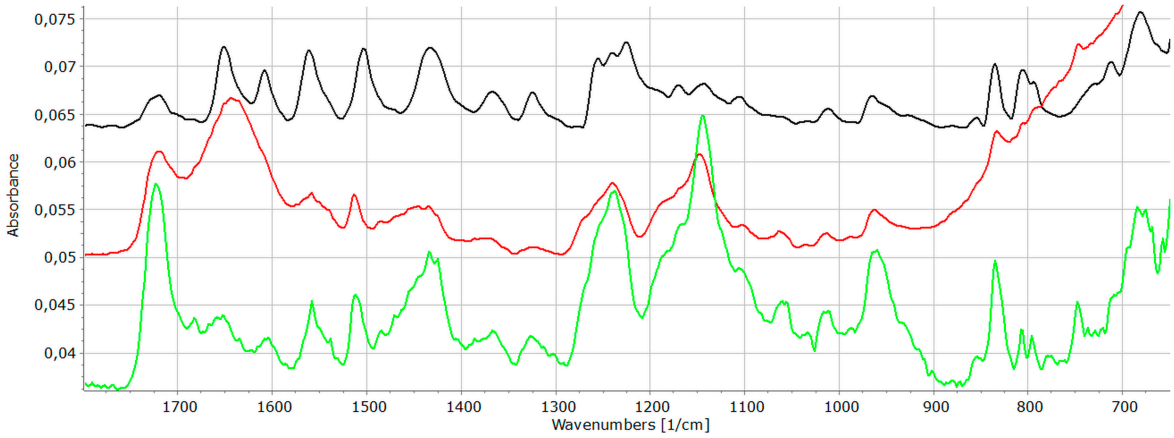

Figure S7. FT-IR spectra of Par-PVC:EE 75:25 samples: original (black), dipped (red), and dried (green).

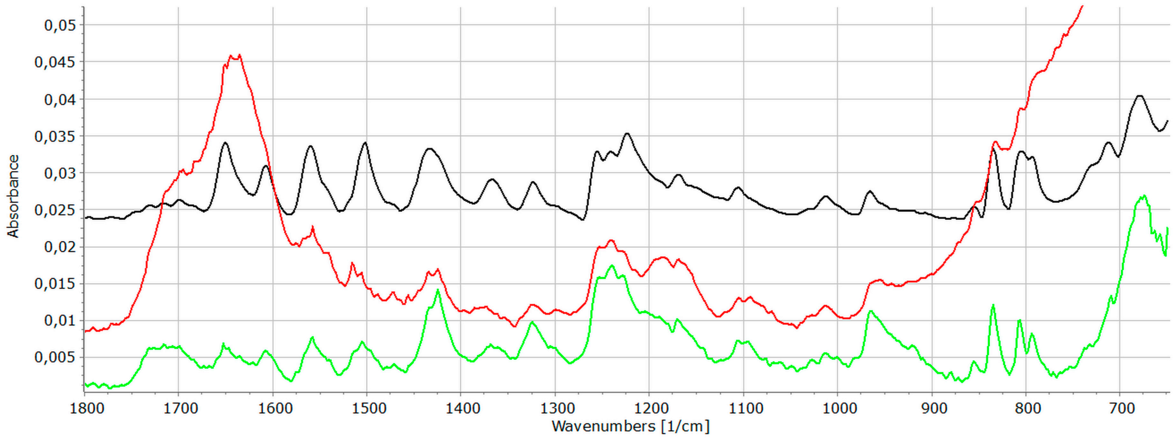

Figure S8. FT-IR spectra of Par-PVC:EL 75:25 samples: original (black), dipped (red), and dried (green).

Supplementary data for 1 week long dissolution study:

Table S2. Release rates and release exponents derived from the Korsmeyer-Peppas model (1-week-long test).

Composition	Eudragit/PVC ratio (%/%)	Pressure (MPa)	R^2	k	n
PAR-PVC-EE	25/75	375	0.9576	4.1992	0.3206
ACE-PVC-EE	75/25	375	0.9918	0.0495	0.532
DIC-PVC-EE	25/75	375	0.8327	3.1193	0.2434
DIC-PVC-EL	50/50	225	0.97	0.2829	0.6428

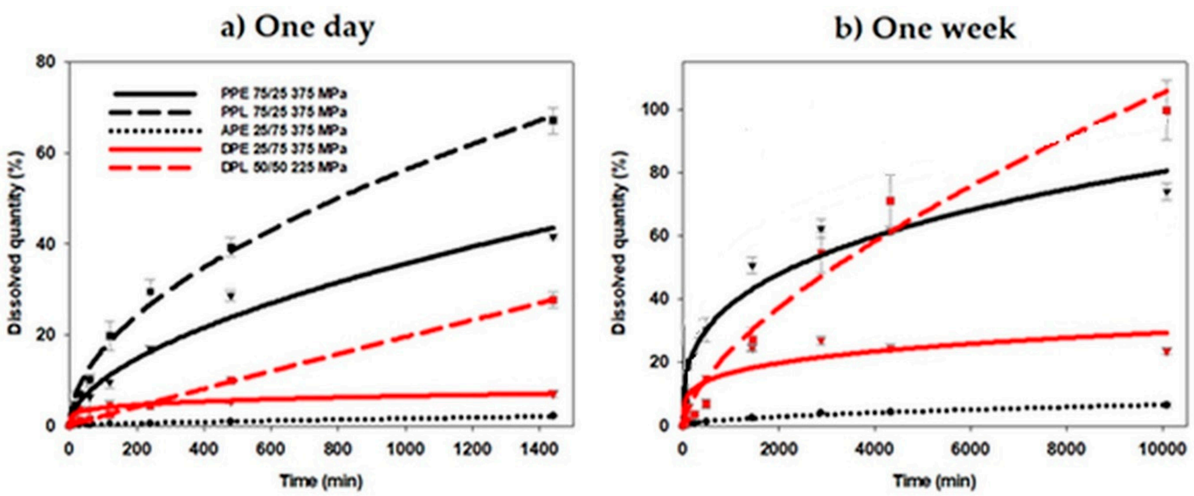

Figure S9. Drug release from various matrices: effect of the drug and polymer properties (a) and effect of the composition and compression force for PAR (b), DIS (c) and ACE (d) containing matrices.

There the results met the expectations, the ACE containing matrix (APE) proved to be the most retentive, liberating around 2% on one day, and reaching 6.5% by the end of a week. As for DIS with EE (DPE), the 24-hour test provided nearly 7% and the one-week test a 23.6% value, while with the EL (DPL) 27.7% was released in one day and 99.85% was reached by the end of the week. PAR with EE (PPE) liberated 42% of the drug during one and 74% in seven days, while if the API was mixed with EL (PPL), the released amount was 67% and 100%, in one day and one week, respectively. Nevertheless, it should be

noted that in this latter case the liberation process reached the maximum 100% till the end of the second day, and after then the drug started to adhere to the silicon tubing of the dissolution device resulting decreasing concentrations, therefore this sample was withdrawn from the one-week dissolution study.

Nevertheless, the results confirm, that strong drug-matrix interactions may help the prolongation of the drug release for several months.

Supplementary Material for ANN modelling:

Table S3. Performance of the neurons.

**Number of hidden neurons**		Approach 1		Approach 2		Approach 3		Approach 4		Approach 5		Approach 6	
		Perf.	Error	Perf.	Error	Perf.	Error	Perf.	Error	Perf.	Error	Perf.	Error
**I+O-3**	Mean	0,8777	0,3995	0,8608	0,3103	0,8357	0,3901	0,9304	107,2409	0,9238	162,4238	0,9293	99,3636
	SD	0,0229	0,0250	0,0368	0,0263	0,0852	0,0538	0,0064	20,2784	0,0039	24,6286	0,0058	11,6035
I+O-2	Mean	0,8832	0,3767	0,8808	0,4548	0,8711	0,3875	0,9240	121,0832	0,9186	154,0890	0,9297	91,7769
	SD	0,0071	0,0959	0,0020	0,0630	0,0264	0,0410	0,0083	18,1608	0,0050	15,8342	0,0018	7,4322
I+O-1	Mean	0,8704	0,3086	0,8959	0,3682	0,8826	0,3459	0,9226	101,3835	0,9230	144,6181	0,9294	88,6256
	SD	0,0293	0,0779	0,0214	0,0978	0,0184	0,0464	0,0075	15,5180	0,0020	10,6401	0,0051	5,0336
I+O	Mean	0,8793	0,4695	0,8825	0,3900	0,8885	0,3806	0,9254	116,9167	0,9181	142,9717	0,9273	92,8801
	SD	0,0151	0,0940	0,0144	0,0939	0,0234	0,0711	0,0078	21,1997	0,0028	8,5369	0,0047	10,5143
I+O+1	Mean	0,8562	0,3349	0,8875	0,4569	0,8839	0,3660	0,9314	118,1403	0,9217	152,1861	0,9281	99,1262
	SD	0,0209	0,0620	0,0062	0,1090	0,0202	0,0445	0,0065	25,2726	0,0017	9,1558	0,0026	4,7649

Table S4. Results of the global sensitivity analysis.

	**Approach 1**		**Approach 2**		**Approach 3**		**Approach 4**		**Approach 5**		Approach 6	
**Modelling type**	**Kinetic based**		**Kinetic based**		**Kinetic based**		**Point-to-point**		**Point-to-point**		Point-to-point	
Input variable	Mean	SD	Mean	SD	Mean	SD	Mean	SD	Mean	SD	Mean	SD
Drug	5,12	3,06					8,21	1,56				
Drug solubility (mg/ml)			1,99	1,94	2,30	2,91			2,11	0,20	2,45	0,22
Drug pKa			1,81	2,17	2,09	2,89			1,41	0,12	1,37	0,10
Excipient	1,05	0,13					1,00	0,00				
Excipient solubility (mg/ml)			1,21	0,49	5,95	26,47			1,53	0,19	1,30	0,13
Excipient pKa			1,40	0,62	15,39	75,30			2,86	0,37	2,52	0,22
Excipient amount (%)	1,02	0,06	1,01	0,06	1,25	0,92	1,62	0,46	1,05	0,07	1,26	0,07
Compression pressure	1,38	0,22	1,46	0,37	1,37	0,94	1,56	0,27	1,25	0,06	1,00	0,03
Hardness					1,80	2,50					1,54	0,20
Porosity					1,28	0,74					1,23	0,05
Peak Shift	5,26	5,24	1,67	0,88	1,87	1,66	1,20	0,13	1,13	0,08	1,20	0,08
